# Probing the time course of head-motion cues integration during auditory scene analysis

**DOI:** 10.3389/fnins.2014.00170

**Published:** 2014-06-24

**Authors:** Hirohito M. Kondo, Iwaki Toshima, Daniel Pressnitzer, Makio Kashino

**Affiliations:** ^1^NTT Communication Science Laboratories, NTT CorporationAtsugi, Japan; ^2^Department of Child Development, United Graduate School of Child Development, Osaka University, Kanazawa University, Hamamatsu University School of Medicine, Chiba University, and University of FukuiSuita, Japan; ^3^Laboratoire des Systèmes Perceptifs, CNRS UMR 8248Paris, France; ^4^Département d'études cognitives, École normale supérieureParis, France; ^5^Department of Information Processing, Interdisciplinary Graduate School of Science and Engineering, Tokyo Institute of TechnologyYokohama, Japan

**Keywords:** auditory streaming, bistable perception, build-up, cocktail party problem, crossmodal, hearing, head movement, virtual reality

## Abstract

The perceptual organization of auditory scenes is a hard but important problem to solve for human listeners. It is thus likely that cues from several modalities are pooled for auditory scene analysis, including sensory-motor cues related to the active exploration of the scene. We previously reported a strong effect of head motion on auditory streaming. Streaming refers to an experimental paradigm where listeners hear sequences of pure tones, and rate their perception of one or more subjective sources called streams. To disentangle the effects of head motion (changes in acoustic cues at the ear, subjective location cues, and motor cues), we used a robotic telepresence system, Telehead. We found that head motion induced perceptual reorganization even when the acoustic scene had not changed. Here we reanalyzed the same data to probe the time course of sensory-motor integration. We show that motor cues had a different time course compared to acoustic or subjective location cues: motor cues impacted perceptual organization earlier and for a shorter time than other cues, with successive positive and negative contributions to streaming. An additional experiment controlled for the effects of volitional anticipatory components, and found that arm or leg movements did not have any impact on scene analysis. These data provide a first investigation of the time course of the complex integration of sensory-motor cues in an auditory scene analysis task, and they suggest a loose temporal coupling between the different mechanisms involved.

## Introduction

The structuring of a sensory scene determines what we perceive: rather than an indiscriminate mixture of acoustic events, a lively conversation between friends can be parsed into meaningful components. The sequential integration and segregation of frequency components for the formation of percepts, which is called auditory streaming, is essential for auditory scene analysis, as sound sources produce information over time. Traditionally, streaming has been studied with a highly simplified experimental paradigm (Miller and Heise, 1950; van Noorden, 1975; see Moore and Gockel, 2012 for a recent review). In such a paradigm, a sequence of two tones, A and B, is presented, with A and B set at different frequencies. The frequency difference between A and B biases the most likely perceptual organization: a small difference favors the perception of one stream, whereas a large separation favors the perception of two streams. Streaming is actually a bistable phenomenon for a range of A and B frequencies (see Schwartz et al., 2012 for a review), as a physically unchanging streaming sequence most often induces successive percepts of one or two streams, in a seemingly random fashion.

In the present study, we focus on the so-called build-up of streaming. This refers to the observation that streaming sequences tends initially to be heard as a single stream (van Noorden, 1975) before bistable alternations begin. The build-up has been widely used to probe streaming in behavior and physiology (e.g., Snyder and Alain, 2007 for a review). Note that, recently, the notion of build-up has been questioned by Deike et al. (2012). They pointed out an important experimental caveat: for build-up to be accurately estimated, the period of time between the onset of the sound and the first subjective report should be treated as missing data, which had not always been the case in previous investigations. When Deike et al. (2012) used a missing-data analysis, they found that build-up was not observed for all frequency separations. However, a build-up was still observed for moderate frequency separations (Deike et al., 2012). Other reports using the missing-data approach and moderate frequency separations also reported a build-up (for instance Pressnitzer and Hupé, 2006; Hupé and Pressnitzer, 2012). We adopted this methodology in the present study.

A last consideration of interest is that streaming may be “reset” by a sudden change in the stimulus. For instance, a change in the ear of entry or in the spatial location of the stimulus (Anstis and Saida, 1985; Rogers and Bregman, 1998) tends to increase the proportion of one-stream reports, as is observed at the onset of a stimulus before build-up occurs. Other manipulations can have the same effect, such as introducing short silent gaps in the streaming sequence (Cusack et al., 2004; Denham et al., 2010) or even engaging and disengaging attention (Best et al., 2008; Thompson et al., 2011). The term resetting suggests that perceptual organization starts anew, but it should be noted that in most experiments the reset was only partial. Furthermore, the actual mechanisms of resetting are unknown. With these qualifications in mind, we will still use the term “resetting” in the following, for consistency with previous reports.

**Figure 1:**
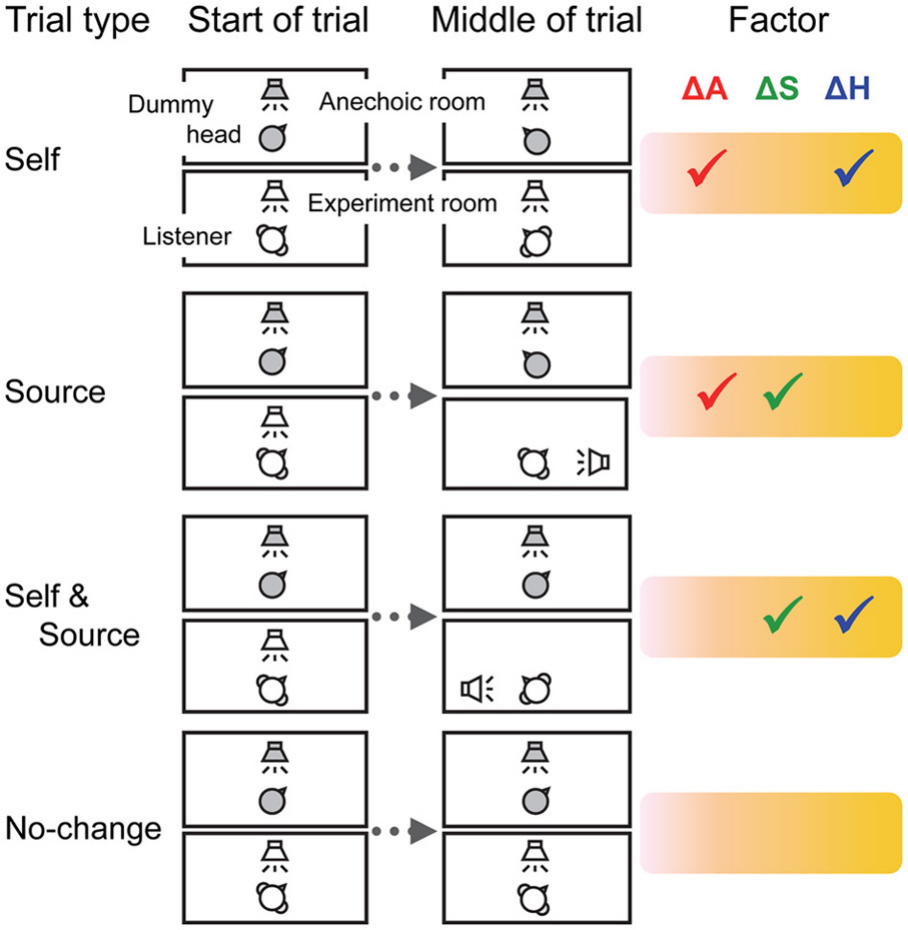
**The experimental setup and trial types**. Auditory stimuli were presented to the Telehead system in an anechoic room. A loudspeaker was positioned in front of the robotic dummy head. Sounds were collected by microphones placed in the dummy head and transmitted to the listener via headphones. The head motion of the listener could be mimicked with minimal latency by the robotic head. Factors: ΔA, changes in acoustic cues at the ears; ΔS, changes in subjective sound localization; ΔH, changes in non-auditory factors related to head motion.

In a previous study, we used the build-up of streaming and its resetting as a tool to investigate the effects of head motion on auditory streaming (Kondo et al., 2012). The rationale was as follows. A change in acoustic cues at the ears, associated with a change in the subjective location of the sound, can induce resetting (Rogers and Bregman, 1998). A voluntary head motion also induces changes in the acoustic cues at the ear, but in theory no sizeable change in subjective spatial location of the sound in allocentric coordinates. What happens to resetting in this case? Perhaps surprisingly, we showed that voluntary head motion did produce some resetting, even though the acoustic scene had not changed (Kondo et al., 2012). Furthermore, we disentangled the various effects of head motion by using a telepresence robot, the “Telehead” system (Toshima et al., 2008). The structure of the various trials types used is summarized in Figure 1 and presented in more details in the Material and Methods section of the present study. In some trials, the Telehead followed head motion, but in others it did not. This allowed to have trials with all possible combinations of three types of cues: (i) changes in acoustic cues at the ears (ΔA), (ii) changes in source location in allocentric coordinates (ΔS), and (iii) changes in non-auditory processes related to head motion (ΔH). A linear model was used to evaluate the effect of each cue. The results showed that all cues impacted perceptual organization, with no counter-balancing between, for instance ΔA and ΔH to avoid a resetting during natural head motion.

In the present study, we reanalyzed the data of Kondo et al. (2012) to focus on the precise time-course of perceptual organization after head motion. It would be possible to hypothesize, for instance, that merging the head position signal with auditory computations is sluggish, leading to the observed lack of exact compensation.

## Material and methods

### Listeners

Ten strongly right-handed listeners were recruited for Experiment 1 (5 males and 5 females; mean age 25.3 years, range 19–30 years). Three different listeners participated in Experiment 2 (2 males and 1 female; mean age 31.3 years, range 24–38 years). All had normal hearing as clinically defined by their audiograms. None had any history of neurological or psychiatric illness or hearing-related disorders. All gave written informed consent, which was approved by the Ethics Committee of NTT Communication Science Laboratories.

### Apparatus

Listeners were seated in the center of a double-walled soundproof room, wearing headphones (HDA 200, Sennheiser). Their head motion was tracked in real time and sent to the Telehead robot, which could mirror the 3D motion with minimal latency and distortion (Toshima et al., 2008). Auditory stimuli were delivered through a loudspeaker (MG10SD0908, Vifa) located 1 m in front of the Telehead dummy head, in an anechoic chamber. Sound was recorded by small microphones (ECM77B, Sony) placed 2 mm inside the entrance of the dummy head's outer ears and transmitted in real time to the headphones. Two light-emitting diodes (LEDs) were used as visual cues to direct head movements and positioned on the left and right sides of the listener at eye level and at a 2-m distance (visual angle re: midline = 60°). The room was darkened for the duration of the experiment.

The Telehead dummy head was made by molding a human head using impression material. The surface was covered with a 1-cm thick layer of soft polyurethane resin. The listeners' head positions were measured with a 3D head-tracker (FASTRAK, Polhemus) placed on the top of the headphones. The position data were obtained at a 120-Hz sampling rate and used to synchronize the yaw, pitch, and roll motions (maximum range 180, 80, and 60°, respectively) of the listener's head with those of the dummy head.

### Stimuli and task procedures

The auditory stimuli were composed of 50 repetitions of a triplet of narrow-band pink noises (roll-off = 3 dB/octave) arranged in an ABA- pattern where A and B represent different noise bands and a hyphen represents a silent interval. The A and B bands were geometrically centered around 1 kHz with a 6-semitone frequency difference between them and a 4-semitone bandwidth. This yielded cut-off frequencies of (749–944) Hz for the A band and (1060–1335) Hz for the B band. The noise bands were generated in the frequency domain and equated in RMS amplitude. The duration of each noise was 62.5 ms, which included rising and falling cosine ramps of 10 ms. The onset asynchrony between successive bands was 100 ms. A background of pink noise was also included to mask any residual line noise of the Telehead system. The pink noise was generated in the frequency domain with cut-off frequencies of (0.1–5) kHz, with a level of −30 dB RMS relative to the A and B bands. The sound pressure level was measured by using ICE couplers with microphones and a measuring amplifier (Brüel and Kjær). The presentation level of the stimuli was set at 65 dB SPL.

Listeners were tested individually. We first explained the concept of auditory streaming by means of a visual illustration of the stimuli. They were instructed to report their percept by pressing one out of two buttons (one-stream when they heard a galloping rhythm ABA-ABA-, or two-stream when they heard A-A-…and –B—B—… each with an isosynchronous rhytm). Listener's responses were held between button presses. Before the first button press, responses were treated as missing data. Listeners were also instructed to move their head to track an LED and maintain it at the center of their gaze. Before the beginning of each trial, they oriented themselves toward the midline and their head positions were calibrated. Then, a blinking LED was randomly presented either on their left or right side and counterbalanced across the trials.

Experiment 1 consisted of four types of trials (Figure 1). In the Self trials, after 10 s of sound presentation, the LED was turned off and another LED was lit on the contralateral side. The listeners were instructed to track this change by moving their head as fast as possible so as to maintain their gaze on the light. The Telehead robot mimicked the head motion, so the Self trials simulated actual head motion. In the Source trials, the LED remained lit on the same side throughout the trial, so that there was no head motion required from the listener. However, the Telehead robot initiated a motion previously recorded from the same listener. This motion had the same acoustic cues at the ears as for the Self trials, but without their motor and volitional components. Such Source trials simulated the displacement of a sound source. In the Self and Source trials, listeners initiated a head motion to follow a change in the visual cue position, but the robot did not move. Such trials have all the motor and volitional components of the Self trials, but without any change in the acoustic cues at the ears. They resulted in an apparent motion of the source in allocentric coordinates, which appeared to follow exactly the orientation of the head (as when one listens to music over headphones). In the No-change trials, the visual cue position was maintained throughout the trial and neither the listener nor the robot moved. The No-change trials were used as a baseline.

Experiment 2 consisted of three types of trials. At the beginning of the Arm trials, an LED was lit on the right side. Listeners were asked to raise their left arm as quickly as possible if the LED was turned off 10 s after stimulus onset. The left arm was chosen as listeners used their right hand to report streaming. An LED on the left side was lit in the Leg trials, and then the listeners raised their left leg if the LED was turned off. The No-change trials were identical to those in Experiment 1 (the LED was maintained lit during the whole duration of the trial).

The trial types were randomly mixed within blocks, for each experiment. The order of the trial types was randomized. In addition to tracking the visual cue, the listeners were instructed to continuously report whether they heard one stream or two.

At least 12 practice trials were run before data collection began. The head movement of the listeners in the final practice trial was recorded to generate the Telehead motion in the Source trials. Experiments 1 and 2 consisted of 6 blocks of 24 trials and 6 blocks of 18 trials, respectively.

### Data analyses

Thirty-six time-series data (temporal resolution, 1 ms) were collected for each trial type. We smoothed the probability of two-stream judgments with 10-ms, non-overlapping rectangular temporal windows (bins). For each bin, we computed a resetting index, *R*, for the Self, Source, and Self & Source trials. *R* was obtained by subtracting the baseline probability of two-stream judgments in the No-change trials to the actual probability of two-stream in the condition of interest. *R* was computed for each bin, trial type *TT*, and listener *L*, as:

(1) $$R_{TT,L}=P_{TT,L}(\text{2 stream})-P_{No-change,L}\left(\text{2 stream}\right)$$

We then built a linear model to estimate the contribution of ΔA, ΔS, and ΔH to resetting. *R* was modeled as:

(2) $$R=K_{A}\Delta A+K_{S}\Delta S+K_{H}\Delta H$$

Three measures of *R* were available for each listener, one for each trial type. For each measure, the values of ΔA, ΔS, and ΔH were set at either 0 or 1 depending on whether the trial type included changes in the corresponding factor (see Figure 1, check marks indicate 1). The system of three equations and three unknowns was then solved for each listener.

We computed *K*_*A*_, *K*_*S*_, and *K*_*H*_ for each time bin from 10 to 20 s after stimulus onset, and performed a repeated-measures analysis of variance (ANOVA) on the values. Tukey honestly significant difference (HSD) tests were used for *post hoc* comparisons (*α*-level = 0.05).

## Results and discussion

We found a build-up pattern for the first 10 s of sound presentation in Experiment 1. The initial report was always one stream and the probability of two streams increased gradually over time. The analysis of interest focused on the percepts reported for the second half of the stimuli, that is, between 10 s and 20 s relative to stimulus onset. The probability of two-stream at 10 s did not depend on trial type. The probabilities of two-stream reports were 61 ± 5, 54 ± 5, 58 ± 5, and 58 ± 5% (means ± *SE*) for the Self, Source, Self & Source, and No-change trials, respectively, *F*_(3, 27)_ = 2.38, η^2^ = 0.03, *p* = 0.09. This indicates, reassuringly, that listeners could not guess the trial types before the presentation of the visual cue.

As just described, at the 10-s point where stimulus manipulation occurred, listeners reported two-stream in roughly 60%, and one-stream in the remaining 40% of trials. According to the standard definition of resetting, a reset implies a switch from two-stream to one-stream. Therefore, according to this definition, resetting could only be measured for those trials where listeners reported two-stream at 10 s. We will term those trials two-stream trials and analyze them separately. However, it could also be that the manipulations had a different effect on perceptual organization, not accounted for by the standard view of resetting: for instance, a change could facilitate a perceptual switch, whatever the state of the listener (one- or two-stream). We thus also applied the same analyses to the one-stream trials, for which listeners reported one-stream at 10 s. In summary, all trials were classified as either one-stream trials or two-stream trials according to the perceptual state of the listener at 10 s. The probability of two-stream at 10 s was normalized to either 0 or 1, respectively, for each type of trials.

**Figure 2:**
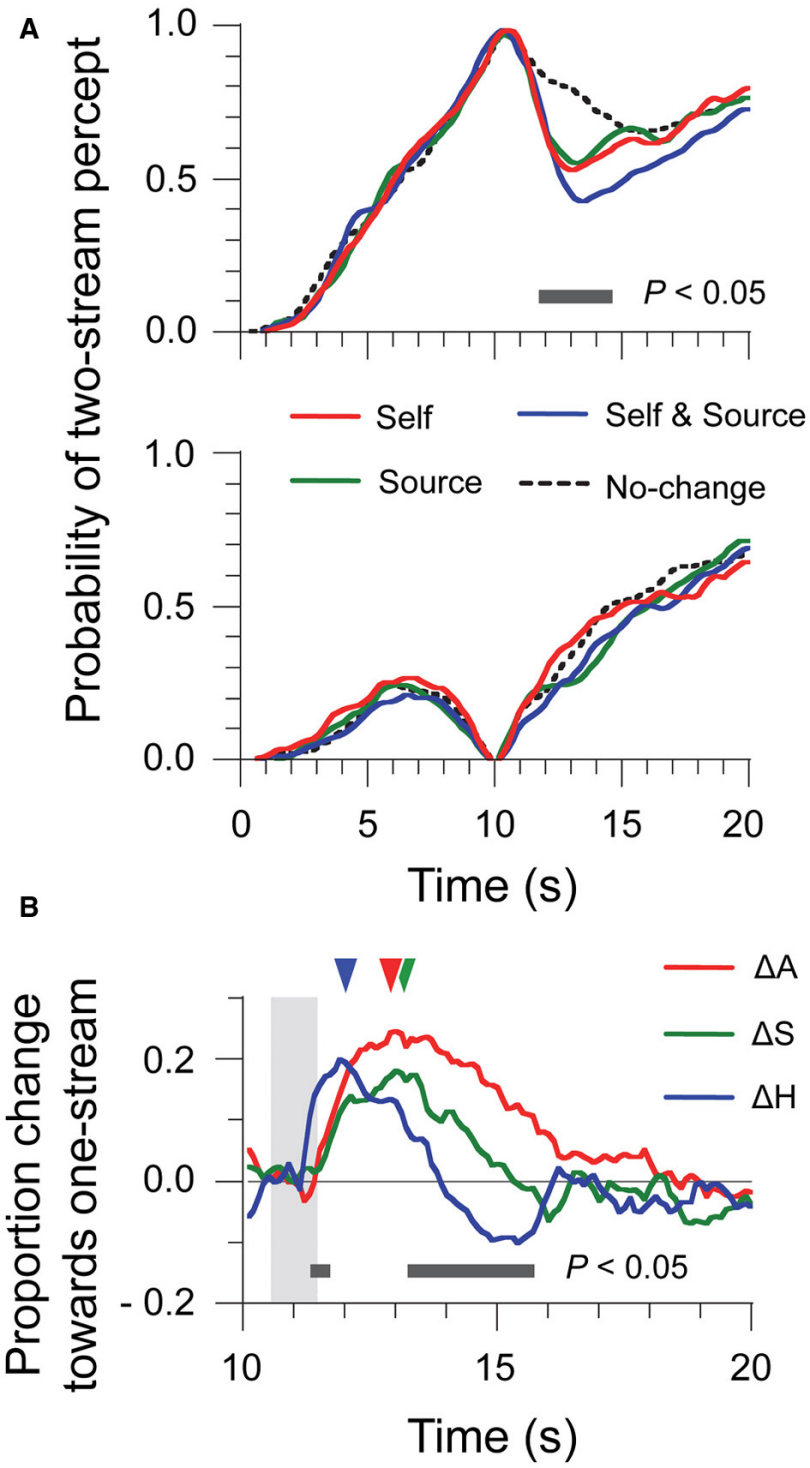
**Time-series data of percept probabilities and factor contributions for Experiment 1. (A)** Normalized data were computed by selecting the trials where perception was either two-stream (top panel) or one-stream (bottom panel) at the 10-s point. **(B)** Contributions of the ΔA, ΔS, and ΔH factors to resetting were estimated for each time bin by means of a linear additive model considering all trial types. The shaded area represents the time window of sound motion and head motion. Triangles indicate the latency of the maximum amplitude for each factor.

The two-stream trials (Figure 2A, top panel) were already analyzed in Kondo et al. (2012) in an a priori time window. Here, using a time-varying analysis and Tukey HSD tests (10-ms time bins, *p* < 0.05 as criterion), we found differences between conditions in a temporal window ranging from 11.7 s to 14.5 s after stimulus onset. For the one-stream trials (Figure 2A, bottom panel), the effect of stimulus manipulation was much less salient. No significant difference was found between any of trial types, at any time bin in the analysis. Thus, head motion and source location changes only affected those trials where perceptual organization was at two-stream at the time of the manipulation.

Because of the lack of effect for the one-stream trials, we computed the time-varying *R* index only for the two-stream trials. Results are shown in Figure 2B, which displays the time-series for the contributions of the ΔA, ΔS, and ΔH factors to *R*. The shaded area in the figure indicates the time interval encompassing the head motion produced by listeners in the *Self* trials: 0.80 s ± 0.07 s, with an onset of motion at 10.6 s. In Experiment 1, the duration of the sound motion was matched with that of head motion (see Material and Methods), so the shaded area also represents the time when stimulus changes were introduced. We compared the contributions for the three factors by a repeated-measures ANOVA. The contribution of ΔH was larger from 11.3 s to 11.7 s but was smaller from 13.2 s to 15.7 s than those of ΔA and ΔS. A further difference is that ΔH produced negative values, that is, a bias toward two-stream, for the later period. The peak amplitudes of the contributions did not differ for different factors: ΔA, 0.26 ± 0.03; ΔS, 0.21 ± 0.02, and ΔH, 0.22 ± 0.03, *F*_(2, 18)_ = 1.71, η^2^ = 0.16, *p* = 0.21. However, the latency to the peak amplitude was earlier for ΔH (12.4 s ± 0.5 s) than for ΔA (13.1 s ± 0.3 s) and ΔS (13.4 s ± 0.4 s), *F*_(2, 18)_ = 3.74, η^2^ = 0.29, *p* < 0.05. So the effects of ΔH on perceptual organization occurred earlier than those of ΔS and ΔA, and also lasted for a shorter time. The initial effect of ΔH was to contribute to resetting, but there was also a “negative” contribution to resetting for ΔH at later times. Such a negative contribution would be what is required for compensating the effects of ΔA and ΔS and prevent resetting when only the head moves and the scene does not change. However, this negative contribution was too slow and too small for canceling out the resetting effects of other factors, like e.g., ΔA in the case of natural head motion.

These new observations on the time-course of ΔH suggest further possible interpretations for the lack of exact compensation between sensory cues and head motion. The ΔH factor reflected the overall effect of head motion, but it could possibly be further decomposed into a volitional component triggering the head movement, a component related to the elaboration of motion commands, and somatosensory feedback information. Obviously, a volitional component would have to be present before any head motion could occur. In addition, it is known that volitional control affects spontaneous switching in auditory streaming (Pressnitzer and Hupé, 2006) as well as in visual bistable stimuli (Meng and Tong, 2004). Therefore, it could be that an early volitional signal could account for the early resetting effect of ΔH, which was only later followed by compensation mechanisms perhaps due to somatosensory feedback.

**Figure 3:**
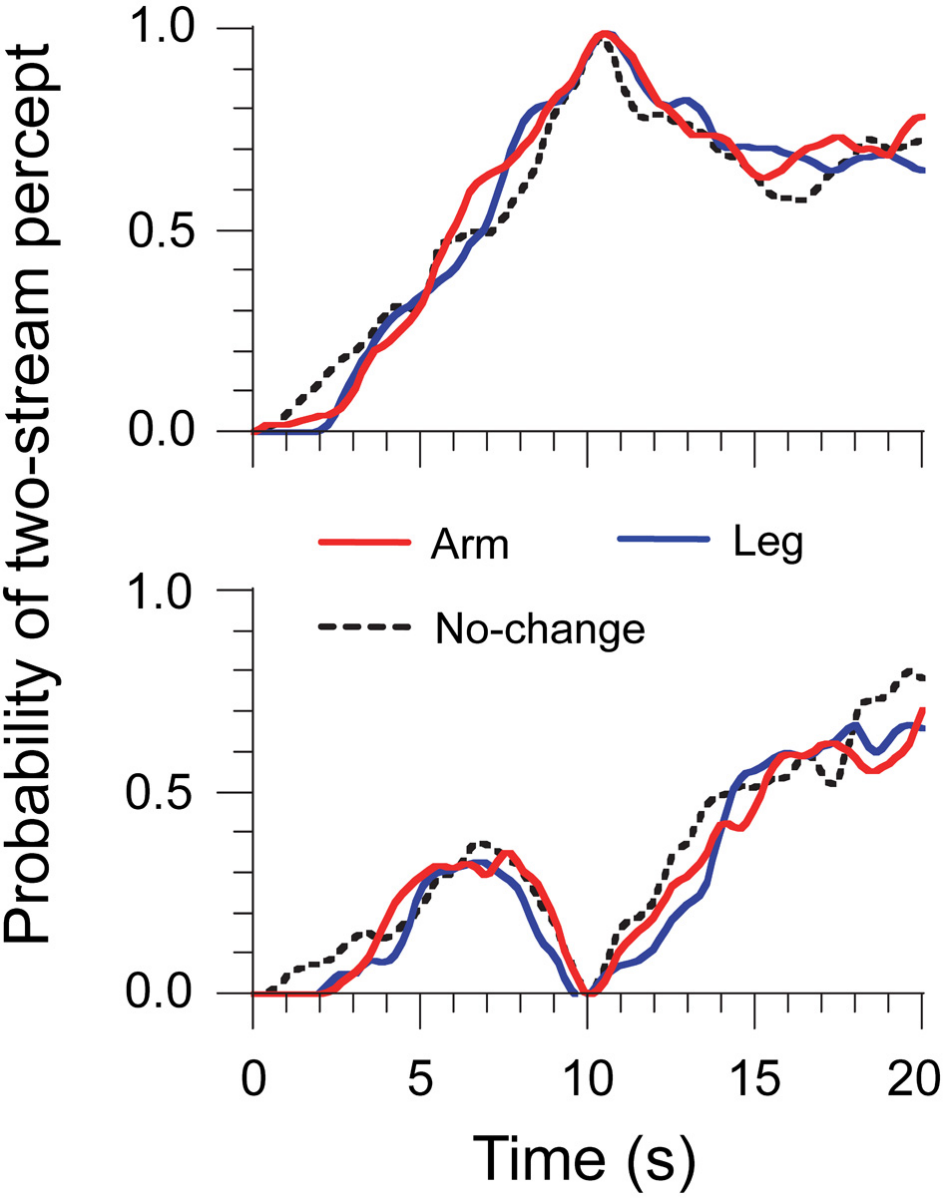
**Time-series data of percept probabilities for Experiment 2**. Normalized data were computed by selecting the trials where perception was either two streams (top panel) or one stream (bottom panel) at the 10-s point.

Experiment 2 aimed at controlling for the volitional component of ΔH. Listeners had to move their arm or leg, but not their head, during the streaming task (see Material and Methods). This task should have a comparable volitional component to the head-motion main task, without any relevant motor or sensory feedback cues for auditory scene analysis. The same analysis of streaming report was used as in the main experiment. Results are displayed in Figure 3. As before, build-up was observed and there was no difference in the probability of two streams at 10 s between the Arm, Leg, and No-change trials: 65 ± 2, 67 ± 2, and 67 ± 3%, *F*_(2, 4)_ = 1.60, η^2^ = 0.10, *p* = 0.31. All the trials were classified under one- and two-stream trials, and the probability of two streams at 10 s was normalized into either 0 or 1. We did not find any difference in the probability of two streams between trial types at any time bin, for either two-stream or one-stream trials. This shows that a volitional component to body motion, as estimated with arm and leg movements, was not a major factor of the pattern of data. It also suggests that the early resetting effect observed for head motion in Experiment 1 was likely not due to volitional anticipation.

To summarize the new experimental findings, we found that acoustic cues (ΔA) and subjective location cues (ΔS) had a sustained and positive effect on resetting. In contrast, the head-motion cues (ΔH) had an early resetting effect followed by a later compensating effect. The early effect did not seem to be related to a volitional anticipation of the motion, as other types of body motion had no effect on auditory streaming.

It may be useful to consider those findings in the light of, on the one hand, the neural bases of sensory-motor integration during head motion, and, on the other hand, the neural bases of auditory streaming. The most obvious impact of head motion on auditory processes is for sound source localization. During head-motion, craniocentric binaural cues such as inter-aural time and inter-aural level differences must be transformed into allocentric coordinates: a stationary source will produce dynamic changes in binaural cues during head motion, changes that must be accounted for by comparing them to those expected because of head motion. This conversion from craniocentric to allocentric coordinates could use efferent copies of the head-motion command, afferent information from neck muscles, or afferent information from the vestibular system (Lewald and Ehrenstein, 1998; Lewald et al., 1999). The precise neural stage at which such signals contact the auditory pathways is not fully known. Human EEG data suggest that the allocentric map may only be fully completed after the primary auditory cortex (Altmann et al., 2009). However, there is also ample evidence from single-unit recordings that motor signals modulate early auditory spatial processing in the inferior (Groh et al., 2001) and superior (Jay and Sparks, 1984; Populin et al., 2004) colliculi, although those studies focused on eye position rather than head position. For auditory streaming, neural correlates are also a matter of debate. Correlates have been claimed at several stages in the auditory pathways: in the auditory cortex (Micheyl et al., 2007), in supra-modal areas such as the intraparietal sulcus (Cusack, 2005), but also subcortically in the auditory thalamus (Kondo and Kashino, 2009), the inferior colliculus (Schadwinkel and Gutschalk, 2011), and even before binaural convergence in the cochlear nucleus (Pressnitzer et al., 2008).

Therefore, a hypothesis to explain the surprising presence of partial perceptual resetting after head motion, even when the scene has not changed, could be that at least parts of network involved in auditory scene analysis are not fully modulated by head position signals during self-motion. This hypothesis would be consistent with findings related to sound source localization, independent of auditory scene analysis (Goossens and Van Opstal, 1999; Vliegen et al., 2004; Altmann et al., 2009). A parallel may exist with vision: eye movements are undoubtedly useful for apprehending a visual scene, but around the time of a saccade the compensation for self-induced motion is far from perfect (Ross et al., 2001). A compression of auditory space has also been reported just before the initiation of rapid head movements (Leung et al., 2008) and during passive body movements (Teramoto et al., 2012). In other words, in this hypothesis, the resetting effect of head motion is not beneficial to auditory scene analysis, but it derives from other constraints on the neural architecture of the system (for instance the difficulty to have precise temporal alignment of all sources of information in two broadly distributed networks). Such an imperfect compensation may have been tolerated by the system as its computational efficiency outweighed any functional disadvantage.

There is another, more speculative interpretation of the observed time-course of head-motion signals on scene analysis. The early component of resetting due to head motion could be related specifically to head-motion volitional signals, anticipating motion and signaling the need to collect novel information (see for instance Kondo et al., 2012, for situations where the head motion disambiguate front/back location cues). In this perspective, at least a partial reconsideration of the current perceptual organization may be useful to integrate as rapidly as possible the new information revealed by head motion.

In any case, our data suggest a temporally-sluggish linkage between scene analysis and sensory-motor integration. Such a loose coupling may reduce the computational demands of combining the two complex functions, without any obvious functional disadvantage (or even a small benefit) in natural auditory scene analysis.

## Author contributions

Hirohito M. Kondo, Iwaki Toshima, Daniel Pressnitzer, and Makio Kashino designed the research; Hirohito M. Kondo, Iwaki Toshima, and Daniel Pressnitzer performed the research; Hirohito M. Kondo, Iwaki Toshima, and Daniel Pressnitzer analyzed the data; and Hirohito M. Kondo and Daniel Pressnitzer wrote the paper.

### Conflict of interest statement

The authors declare that the research was conducted in the absence of any commercial or financial relationships that could be construed as a potential conflict of interest.
